# Geographic distribution and impacts of climate change on the suitable habitats of *Rhamnus utilis* Decne in China

**DOI:** 10.1186/s12870-023-04574-4

**Published:** 2023-11-27

**Authors:** Song Guiquan, Feng Jiali, Gong Shuai, Hao Wenya, Kong Xiangkun, Zhao Sheng, Zhao Yueling, Jiang Xuelian

**Affiliations:** 1 Weifang Municipal Key Laboratory of Agricultural Planting Quantization and Application, Weifang University, Weifang, Shandong 261061 China; 2 Sinochem Agriculture Holdings Co. Ltd, Beijing, 1000323 China

**Keywords:** Global climate change, *Rhamnus utilis*, Species distribution model, Maxent, Potential suitable habitat

## Abstract

**Background:**

*Rhamnus utilis* Decne (Rhamnaceae) is an ecologically and economically important tree species. The growing market demands and recent anthropogenic impacts to *R. utilis* forests has negatively impacted its populations severely. However, little is known about the potential distribution of this species and environmental factors that affect habitat suitability for this species. By using 219 occurrence records along with 51 environmental factors, present and future suitable habitats were estimated for *R. utilis* using Maxent modeling; the important environmental factors affecting its distribution were analyzed.

**Results:**

January water vapor pressure, normalized difference vegetation index, mean diurnal range, and precipitation of the warmest quarter represented the critical factors explaining the environmental requirements of *R. utilis*. The potential habitat of *R. utilis* included most provinces from central to southeast China. Under the climate change scenario SSP 245, Maxent predicted a cumulative loss of ca. 0.73 × 10^5^ km^2^ in suitable habitat for *R. utilis* during 2041–2060 while an increase of ca. 0.65 × 10^5^ km^2^ occurred during 2081–2100. Furthermore, under this climate change scenario, the suitable habitat will geographically expand to higher elevations.

**Conclusions:**

The findings of our study provide a foundation for targeted conservation efforts and inform future research on *R. utilis*. By considering the identified environmental factors and anticipating the potential impacts of climate change, conservation strategies can be developed to preserve and restore suitable habitats for *R. utilis*. Protecting this species is not only crucial for maintaining biodiversity but also for sustaining the economic benefits associated with its ecological services.

## Introduction

Climate is one of the most crucial elements affecting plant life cycles and fitness [1, 2], niche construction [3, 4], species coexistence [5], community composition [6], and the geographic distribution of species [7, 8]. The Fifth Assessment Report of the IPCC states that global climate warming will continue indefinitely, and by 2100s the mean temperature of the Earth will increase by 0.3–4.5 °C compared with that during 1986–2005 [9]. Changes in the effects of climate change on the world’s biota are creating a critical issue for scientists, conservationists, and decision-makers [10]. Plant species are unlikely to be able to migrate sufficiently quickly. They will, therefore, have to respond in situ, e.g. through local adaptation, which may often fail [11]. Therefore, in order for forest managers to assess the susceptibilities of ecosystems and species to climate change, it is critical to study how climate change affects the spatial distribution of species on the landscape.

One method of predicting the suitable geographic range and ecological needs of a species is a Species Distribution Model (SDM) [8]. There are various kinds of SDMs, such as CLIMEX [12], maximum entropy (Maxent) [7], Genetic Algorithm for Rule-set Production [13–15], Ecological Niche Factor Analysis [16], and bioclimate envelope [17], which are used to forecast the geographic distribution, ecological reactions, and ecological needs of various species. Among the various species distribution models, Maxent stands out as a leading choice for several compelling reasons. This general-purpose machine learning method is designed to require only information on the presence of species, not their absence, a feature that elegantly solves the common problem of gathering absence data [18]. Maxent’s predictive capability is renowned for its accuracy, robustness, and efficiency, often considered unparalleled in the field of habitat modeling [19]. Its ability to handle both linear and nonlinear relationships between species and environmental factors allows for a more nuanced understanding of species’ ecological niches. Furthermore, Maxent’s flexibility in accommodating various types of data, its resilience to small sample sizes, and its powerful algorithms for identifying key environmental predictors make it a preferred tool for ecologists and conservationists [20].

*Rhamnus utilis* Decne (Rhamnaceae) is an ecologically and economically important shrub species occurring in forests, thickets, mountains, and hills below an elevation of 3300 m [21]. Based on the description in Flora of China, this species occurs in more than 15 provinces in southern China [21], and it thrives on light sandy to medium loamy soil that is moist and well-drained and has an important effect on boosting the water retention capacity of soils and decreasing soil loss to surface runoff [21]. The fruits of *R. utilis* are a valuable resource, containing high levels of protein and a yellow pigment that is used in the manufacture of lubricating oil, printing ink, and soap [22, 23]. Furthermore, several pharmaceutical research studies have shown that the bark, leaves, and seeds of this species have anti-inflammatory, and anti-allergic properties [24, 25].

Given the economic and ecological importance of this species, understanding how a changing climate will affect its favorable habitat is critical. Shifts in temperature, precipitation, and other climatic factors can have profound impacts on the species’ survival and distribution, altering the availability of resources, affecting reproductive strategies, and influencing interactions with other species within the ecosystem. Nevertheless, the current understanding of the suitable geographic range of this species has been limited to the records in Flora of China, which only documents the provinces without specific distribution locations or the specific environmental conditions that influence habitat appropriateness.

In the present study, Maxent modeling was used to project the potential geographical ranges of this species by using database recent historical and geo-referenced occurrence records as well as to evaluate habitat suitability and important environmental factors that shape its distribution. The objectives of the present study include: (1) modeling the potential suitable habitat for this species under current and further climate change scenarios, (2) identifying important environmental factors that shape its distribution; and (3) using projected future climate conditions to quantify the changes in its geographical ranges in a way that will help researchers to create suitable habitat.

## Results

### Current potential habitat and model accuracy

**Fig. 1:**
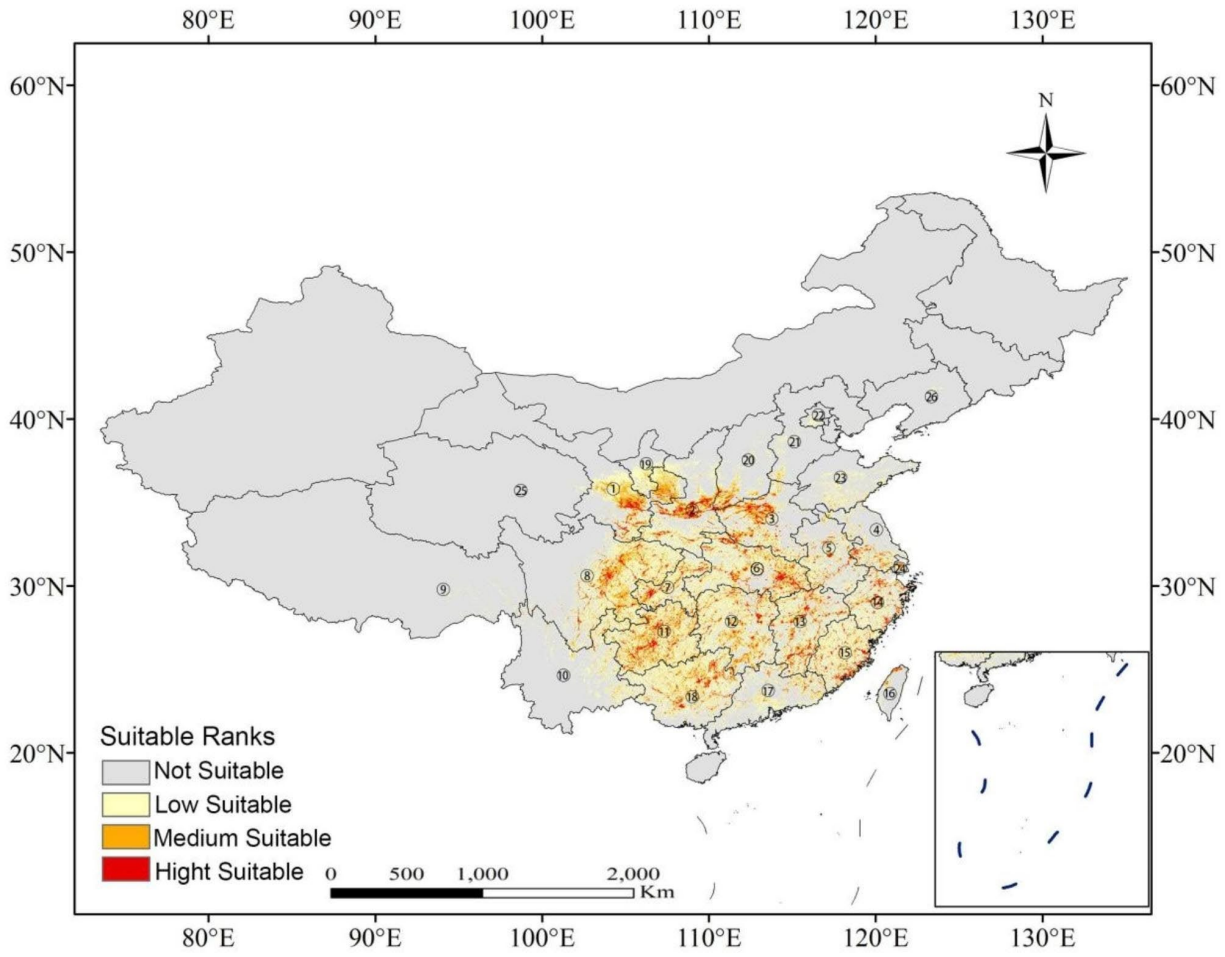
Predicted potential distribution map of *Rhamnus utilis* Decne under current climate scenario. 1. Gansu; 2. Shaanxi; 3. Henan; 4. Jiangsu; 5. Anhui; 6. Hubei; 7. Chongqing; 8. Sichuan; 9. Tibet; 10. Yunnan; 11. Guizhou; 12. Hunan; 13. Jiangxi; 14. Zhejiang; 15. Fujian; 16. Taiwan; 17. Guangdong; 18. Guangxi; 19. Ningxia Hui Autonomous Region; 20. Shanxi; 21. Hebei; 22. Beijing; 23. Shandong; 24. Shanghai; 25. Qinghai; 26. Liaoning

The potential distribution of *R. utilis* was accurately predicted by the Maxent model as evidenced by high AUC values of 0.978 ± 0.001 (training) and 0.959 ± 0.012 (testing), and TSS scores of 0.865 ± 0.025 (training) and 0.842 ± 0.034 (testing). Areas in provinces such as southeastern Gansu, southern Shanxi, eastern and central to western Sichuan, Sichuan, northwestern Henan, western Chongqing, eastern Hubei, southern Jiangxi, southeast Fujian, central to northern Zhejiang, and northern Taiwan were appropriate for the growth of *R. utilis* (Fig. 1). The current areas of moderately and highly suitable habitat for *R. utilis* encompasses ca. 3.16 × 10^5^ and 1.18 × 10^5^ km^2^, respectively, accounting for 3.41% and 1.28% of China’s land area.

### Important environmental factors

Based on the contributions of the various environmental factors to the Maxent model (Table 1), the main factors contributing to the *R. utilis* distribution model were VAPR01 (37.7% of variation), NDVI (27.3%), BIO2 (15.0%), and BIO18 (4.5%) (Table 1), yielding a cumulative contribution as high as 84.5% (Table 1). In contrast, additional factors had smaller contributions, indicating their restricted impact on the distribution of appropriate *R. utilis* habitat (Table 1).

**Table 1:** Percentage contributions and permutation importance of the variables included in the Maxent models for *Rhamnus utilis*

Code	Environmental Variables	Contribution%	Permutation importance
VAPR01	Water vapor pressure of January	37.7	32.8
NDVI	Normalized difference of vegetation index	27.3	25.5
BIO2	Mean diurnal range	15	11.7
BIO18	Precipitation of warmest quarter	4.5	10.3
SLOPE	Slope degree	4.3	3.1
VAPR07	Water vapor pressure of July	2.4	2.2
ASPECT	Aspect	1.8	1.6
ELEV	Elevation	1.7	5.8
SRAD11	Solar radiation of November	1.2	1.5
BIO14	Precipitation of driest month	0.7	1.5
BIO3	Isothermality	0.7	0.6
CLAY	Soil clay percentage	0.5	0.6
SAND	Soil sand percentage	0.5	1
SRAD4	Solar radiation of April	0.5	0.1
BIO7	Temperature annual range	0.4	0.4
SRAD8	Solar radiation of August	0.4	1
SRAD9	Solar radiation of September	0.3	0.2
BUCK	Soil buck density	0.2	0.2

**Fig. 2:**
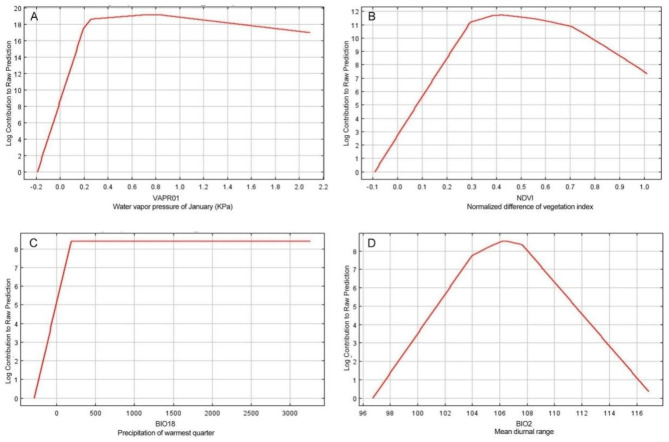
Response curves for important environmental predictors in the species distribution model for *Rhamnus utilis*

The response curves (marginal responses generated through keeping the remaining bioclimatic factors in the mean sample values) of the four critical factors in Maxent for examining the *R. utilis* climatic preference are shown in Fig. 2. Overall, VAPR01 and BIO18 showed a nonlinear logistic response pattern, with the probability of presence increasing when VAPR01 < 0.2 KPa and BIO18 > 250 mm; in contrast, NDVI and BIO2 both showed an increasing and then decreasing pattern, with the maximum probability of presence occurring at 0.4 and 10.6 °C (Fig. 2).

### Future changes in suitable habitat area

**Fig. 3:**
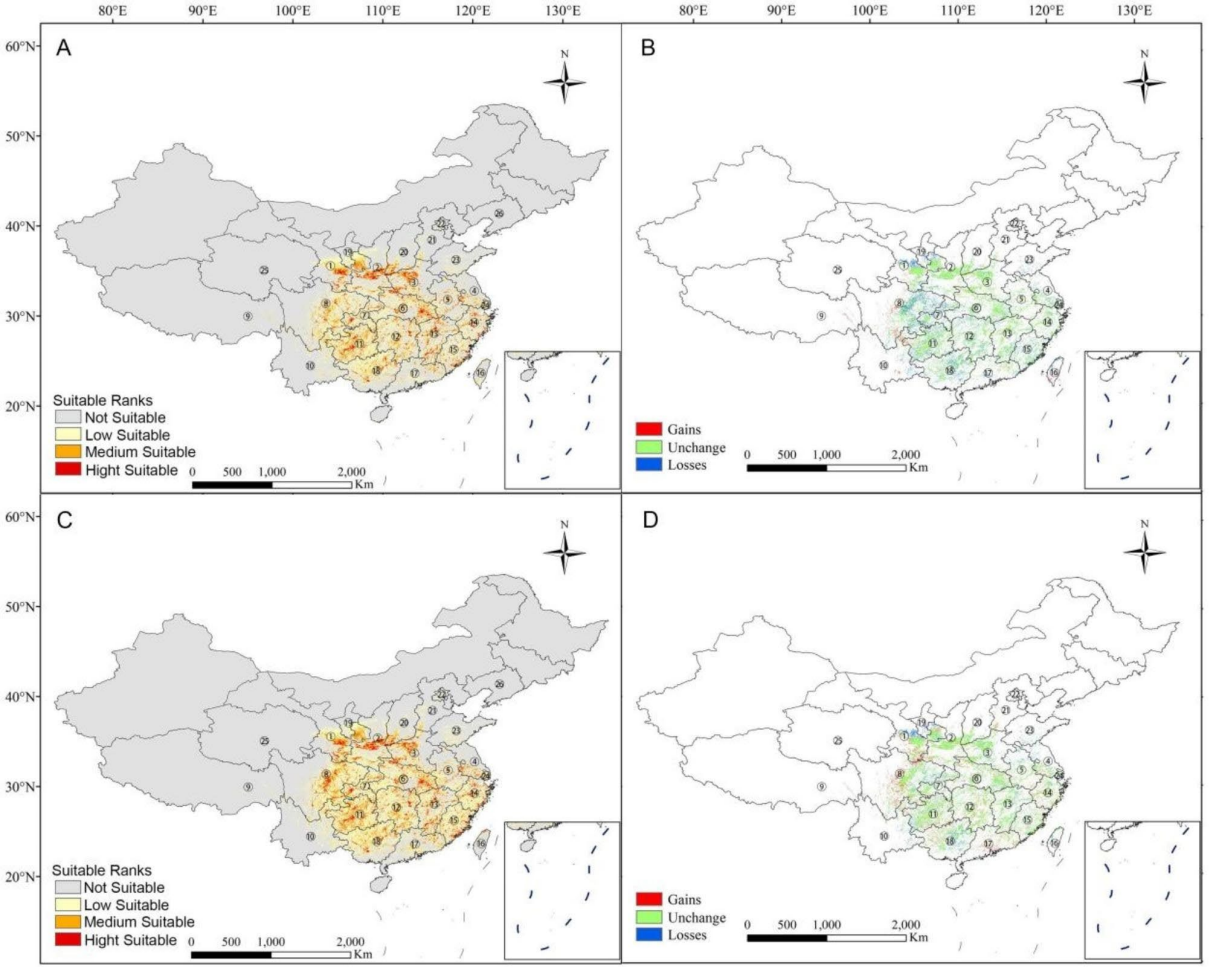
Predicted potential distribution map of *Rhamnus utilis* under future climate change scenario. 1. Gansu; 2. Shaanxi; 3. Henan; 4. Jiangsu; 5. Anhui; 6. Hubei; 7. Chongqing; 8. Sichuan; 9. Tibet; 10. Yunnan; 11. Guizhou; 12. Hunan; 13. Jiangxi; 14. Zhejiang; 15. Fujian; 16. Taiwan; 17. Guangdong; 18. Guangxi; 19. Ningxia Hui Autonomous Region; 20. Shanxi; 21. Hebei; 22. Beijing; 23. Shandong; 24. Shanghai; 25. Qinghai; 26. Liaoning

Under the SSP245 climate change scenario, Maxent predicted that R. *utilis* would lose a cumulative amount of approximately 0.73 × 10^5^ km^2^ in suitable habitat area during 2041–2060. This loss mainly occurred in Guangxi, Guangdong, Fujian, eastern Sichuan, southern Gansu, and the Ningxia Hui Autonomous Region (Fig. 3). In contrast, under the same climate change scenario and during 2081–2100, Maxent predicted a cumulative gain of approximately 0.65 × 10^5^ km^2^ in suitable habitat area. The increase mainly occurred in central Sichuan Province; at the same time, Maxent predicted an area loss of approximately 0.08 × 10^5^ km^2^ in southern Gansu and the Ningxia Hui Autonomous Region. Upon careful examination, we found that the areas of increase were mainly concentrated in high-altitude regions, while the areas of decrease were primarily located in low-altitude regions.

## Discussion

Understanding the distribution of a species is one of the prerequisites for employing it during ecosystem restoration [7, 14, 17]. Although *R. utilis* is a fast-growing species that has been frequently applied in the ecological rehabilitation of temperate forests and riverbanks in eastern and central China, the influences of alterations in climate on its distributions have never been evaluated. Our current work modeled the distributions of *R. utilis* in the present and future climatic contexts, and thus will provide a reference for using this species to allowing land managers to develop improved forest management practices and species protection strategies.

### Distribution and prediction of *R. utilis*

Our results demonstrate that *R. utilis* has an extensive current distribution, with suitable habitat existing in the provinces of Jiangsu, Jiangxi, Shaanxi, Shanxi, Sichuan, Fujian, Gansu, Guizhou, Hebei, Henan, Hubei, Hunan, and Zhejiang. The results of our model suggested that under present climate conditions, moderately and highly suitable habitat for *R. utilis* spanned around 14.82 × 10^5^ km^2^. Our results are generally consistent with previous reports in Flora of China [21]. In addition, some areas of Yunnan, and Ningxia Hui Autonomous Region were also shown to be suitable for this species. However, it is important to note that these predictions do not necessarily indicate that these places are suitable for its growth, and further studies with relevant field verification would be needed to confirm this conclusion.

### Environmental factors affecting *R. utilis* distribution

Factors affecting the geographic distribution of species are a critical issue in ecology and evolution. Of the 18 environmental factors incorporated into our model, VAPR01, NDVI, BIO2 and BIO18 (Table 1) yielded cumulative contributions of as high as 84.5%. However, it’s important to acknowledge that when creating the response curve, all bioclimatic variables, except for the focal factor, were maintained at the average sample value (i.e., other variables were kept stable). In reality, these variables do not remain fixed at their average values. Changes in the appropriateness of responses due to interactions between these factors can take unexpected directions that marginal response curves might not fully reveal. This is because natural conditions don’t keep other factors at their averages; they are influenced by various interactions. Nevertheless, our method enabled us to explore the relationships between specific factors and the likelihood of identifying suitable habitat [15]. The probability of presence of *R. utilis* peaked when VAPR01 ≥ 0.2 KPa. This means that the plant is able to grow and survive best in areas with relatively high humidity. Water vapor pressure is a measure of the amount of water vapor in the air, and it plays a critical role in determining the overall moisture content of an ecosystem [26]. For *R. utilis*, a water vapor pressure of 0.2 KPa or greater POSSIBLY COULD provides the ideal level of humidity for the plant to grow and thrive [26]. This can help to ensure that the plant has access to the moisture it needs to survive, while also providing the ideal conditions for photosynthesis, growth, and reproduction [26, 27].

The effect of vegetation indices, i.e. NDVI, also indicated these indices have a significant contribution to the existence of *R. utilis*, demonstrating that NDVI has the capacity to influence the distribution of *R. utilis*. This might be due to the fact that the majority of *R. utilis* populations are found in forests, thickets, mountains, and hills below an elevation of 3300 m [21]. In addition, past research has shown that a link exists between NDVI and features of the canopy including net primary production [28], the percentage of absorbed photosynthetically active radiation [29], the leaf area index [30], and evapotranspiration [30, 31]. However, the habitat parameters will need to be studied in detail in future to draw a clear-cut conclusion.

BIO2 (mean diurnal range) and BIO18 (precipitation of warmest quarter) also are important environmental factors that affect the presence of *R. utilis.* The mean diurnal range (BIO2) indicates the variation in temperature over a day, and changes in temperature may have a significant impact on plant growth, especially photosynthesis and respiration of the plant, contributing to nutrient buildup [32]. In addition, drought stress has been reported to result in a significant decrease in plant height, leaf area, the number of branches produced, and photosynthesis of *R. utilis.* [33] Furthermore, water availability may directly affect the emergence and development of seedlings [34]. To mitigate the negative impact of drought stress on *R. utilis*, it is important to manage water resources in a sustainable manner and to conserve natural water sources that support the growth of this species. Additionally, efforts to enhance the drought tolerance of *R. utilis*, such as through breeding programs or the use of drought-tolerant cultivars, can help to ensure its persistence in areas that are prone to drought.

### Impact of climate change on the distribution of *Rhamnus utilis* Decne and related forest ecosystems

Global warming will result in some species migrating to high latitudes or elevations [7, 15], while other species may use physiology or phenology adapt to climate change [35]. Our study adopted the climate change scenario SSP 245. This scenario is an intermediate scenario that assumes that CO_2_ emissions begin to decline in 2045, and by 2100, they will be virtually half of what they were in 2050. The SSP 245 scenario also requires a peak in methane (CH_4_) emissions by 2050 and that they should then decline to about 75% of 2040 levels; meanwhile, by 2040 sulphur dioxide (SO_2_) emissions should fall to around 20% of 1980–1990 levels [See ref [36] and references therein]. Our result predicted that, during 2041–2060, the cumulative loss of appropriate habitat for *R. utilis* should be ca. 2.0 × 10^5^ km^2^ compared to current conditions. Consistent with former studies (e.g. ref. 37,38), the lost areas would mainly occur at low elevations, with the increased areas would mainly occur at high elevations. Such result may indicate *R. utilis* would be unable to tolerate continuously increasing temperatures. Furthermore, changes of precipitation and temperature regimes may induce a phenological shift of *R. utilis* species, thus having indirect effects on the dependent flora and fauna. Additionally, such alterations would adversely affect numerous terrestrial insects, birds, and mammals with a direct or indirect dependence on *R. utilis* seeds, flowers, and fruits. In contrast, during 2081–2100, Maxent predicted the cumulative gains for *R. utilis* would be ca. 0.73 × 10^5^ km^2^ in appropriate habitats compared to current conditions. This, as state above, may be due to the scenario we selected, i.e. a predicted decline in CO_2_ and CH_4_ in the 2100s.

### Implications for conservation plans

According to our model predictions, the potential suitable habitat of *R. utilis* increased in high elevations under future climate scenarios. Ensuring continuous monitoring and regular updating of climate models is essential for the accuracy and sensitivity of these predictions. We propose that *R. utilis* plantations in appropriate habitats could serve as a preservation strategy, allowing the species to respond to future climate change. In addition, our findings might be used to categorize natural habitats of *R. utilis*, ranging from low to high risk for this species, based on predicted climate change during conservation planning, In other words, we should only introduce this species in areas of suitable habitat. Moreover, natural regeneration must be maximally conserved in high-risk regions under the future climate scenarios. The unchanged appropriate habitat may provide underlying refugia for climate change, and preserving these habitats is an essential aspect of the conservation and protection of in-situ and ex-situ *R. utilis* forests. To further mitigate the effects of climate change on its habitat, establishing ecological corridors can be used to ensure natural dispersal and gene flow of R. utilis. Finally, flexibility and adaptability must be maintained within conservation plans, allowing for potential future changes and involving periodic review and adjustment of conservation strategies and measures.

### Limitations of modeling and future research directions

Species distribution modeling has been widely adopted and has been proven to be an effective method for providing related guidelines for forest management under future global climate change [13, 14, 17]. However, there are uncertainties in the use of different climate change scenarios for projecting possible plant distribution. Furthermore, the BCC-CSM2 model was used in the present work, but it showed the uncertain nature of future climate change, even though it is recommended for studying climate change in China, thus leading to uncertainties in projected habitat distribution/suitability. Consequently, future studies must adopt diverse SDMs and GCMs. Additionally, although Maxent models are commonly used, there exist several restrictions that should be highlighted and thoroughly considered [37]. The current study complies with the need for presence-only data derived from a variety of diverse and multifaceted sources. However, the collected data may not be fully representative, leading to potential biases [38]. Nevertheless, the sampling bias layer used in our models reflects only a near approximation of actual species distribution. In addition, some biologically important factors, such as human activities, dispersal capability, and competition, were not included in the model because robust data were lacking. This omission points towards an opportunity for future research to incorporate these elements, ensuring a more comprehensive and realistic representation of species distribution dynamics. Finally, future research should be integrated with field studies to validate our results, allowing for a more nuanced understanding of the distribution patterns and facilitating the application of our findings in conservation and management efforts.

## Conclusions

Our results indicate that January water vapor pressure, the normalized difference vegetation index, mean diurnal range, and precipitation of the warmest quarter represented the critical factors explaining the environmental requirements of *R. utilis*. The potential habitat of *R. utilis* included most provinces from central to southeast China. Under climate change scenario SSP 245, Maxent predicted a cumulative loss of ca. 0.73 × 10^5^ km^2^ of suitable habitat for *R. utilis* by 2041–2060, while an increase of ca. 0.65 × 10^5^ km^2^ occurred in the 2081–2100. Furthermore, under this climate change scenario, the suitable habitat will geographically expand to higher elevations. Our results will assist land managers in avoiding the blind introduction of *R. utilis* into unsuitable habitat and enhance its quality and production.

## Methodology

### Species distribution data

**Fig. 4:**
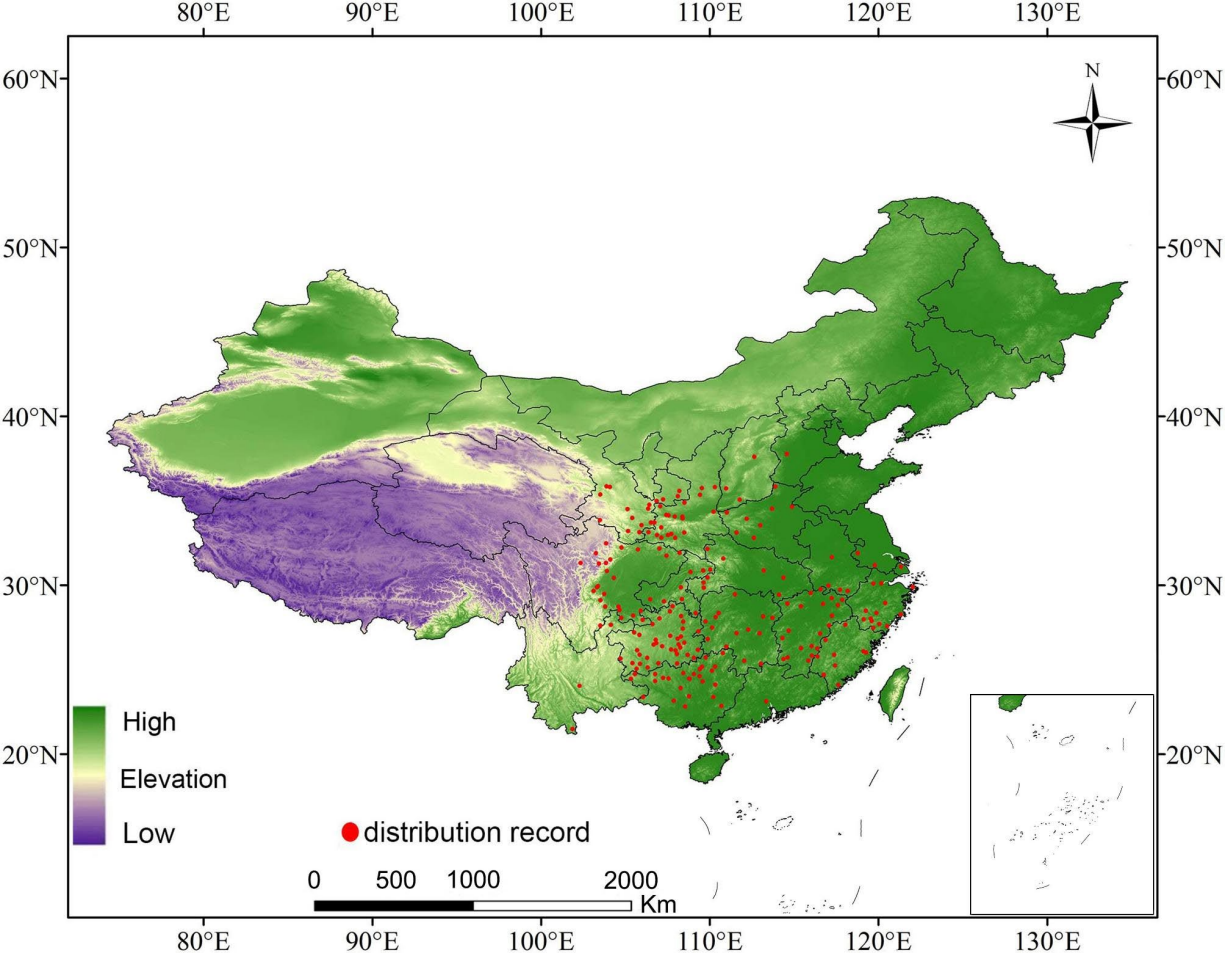
Distribution records of *Rhamnus utilis* Decne in China

The species occurrence data of *R. utilis* were gathered from the online herbaria databases shown below: the Chinese Virtual Herbarium (http://v5.cvh.org.cn/), Tropicos (http://www.tropicos.org/), and the GBIF (http://www.gbif.org). Inaccurate locations without precise geo-coordinates in the occurrence records were excluded. For specimens with only village locations documented in the Chinese Virtual Herbarium, we identified their longitude and latitude via Google Earth (http://ditu.google.cn/). Duplicate data points were eliminated, and the remaining points were subjected to spatial filtering. Hence, a maximum of one point per 1.0 × 1.0 km grid cell was mapped. The total number of geo-referenced occurrence records used was 219. (Fig. 4).

### Environmental variables

The 54 environmental factors that can potentially affect the distribution of *R. utilis* were used. Those include 19 bioclimatic along with 12 solar radiation and water vapor pressure variables acquired from the World Climate Database [39]. Data related to three topographic variables of slope, aspect, and elevation covering 1984–1995were acquired from the RESDC website (http://www.resdc.cn/Default.aspx). Also, data for seven soil variables including soil organic carbon content, soil pH, soil bulk density, soil conductivity, along with soil sand, silt and clay percentage were obtained from the Harmonized world soil database v1.2 [40]. Moreover, normalized difference of vegetation index (NDVI) data were acquired from the China Meteorological Data Sharing Service System.

With regard to future climate scenarios, the climate change modeling data from the Beijing Climate Center climate system model version 2 (BCC-CSM2)-MR of the CMIP6 model were adopted from the Shared Socio-economic Pathways (SSPs) 245 scenario, as put forward by the Intergovernmental Panel on Climate Change to predict the distribution of *R. utilis* in 2041–2060 and 2081–2100. The suppliers of BCC-CSM2 advised it should be used for research related to short-term operational climate prediction and change within China [see ref [41]. and references listed therein]. The SSP 245 scenario indicates possible radiative forcing in 2100 relative to the optimistic + 4.5 W/m^2^ pre-industrial value [42]. The selection of SSP245 was based on its representation as an intermediate scenario. This scenario provides a balanced view of the future, considering both mitigation policies and socio-economic changes. It allows us to explore the potential impacts on the species’ distribution without leaning towards extreme pessimistic or optimistic views. By choosing the time periods ‘2041–2060’ and ‘2081–2100,’ we intend to investigate two separate future scenarios, providing a substantial time gap to enable the observation of potential shifts and trends in the species’ distribution over the medium to long term. Other variables, such as soil and topography, were included in our analysis of the current distribution of *R. utilis*. However, when modeling the future potential habitat, future values for these variables should ideally be used [38]. Since expected changes in these variables were not available for modeling within future climate change scenarios, we left them unchanged in our predictions [38]. Therefore, those variables were left unchanged for the current analysis of the future potential suitable habitat for *R. utilis*. To ensure consistent results among diverse layers, we processed all environmental layers with identical cell size, spatial extent, while using WGS84 projection in the ArcGIS 10.0 scenario.

To reduce collinearity and minimize model overfitting, we applied Principal Component Analysis (PCA) in conjunction with Pearson correlation analysis. If a pair of variables had a correlation coefficient greater than |0.85|, they were considered proxies of one another, and one of the variables was consequently removed from the analysis. Factors enrolled into the final environmental dataset used here included April and August solar radiation (SRAD4 and SRAD8), aspect, average diurnal temperature range (BIO2), elevation, isothermality (BIO3), January, July, and November water vapor pressure (VAPR01, VAPR7, and VAPR11, respectively), NDVI, precipitation of driest month (BIO14), precipitation of warmest quarter (BIO18), slope, soil buck density, soil sand and clay content, and annual range of temperature (BIO7) (Table 1).

### Model simulation and evaluation

Models were established with Maxent version 3.3.3 k [43] based on species records together with bioclimatic variables. We used 25% and 75% of occurrence records for model testing and training, respectively. It is well known that sampling bias significantly affects presence-background distribution models, which was avoided by using one bias file layer in the present work [44]. A bias file layer was produced based on occurrence point through the derivation of one Gaussian kernel density map according to the description by Elith et al. [19]. We used a bias file in Maxent to create maps. Recent studies have indicated that the default setting of Maxent may not necessarily be suitable at all times, especially if only a few species occurrence records can be obtained [20]. As a result, we evaluated various regularization multiplier values, taking into account feature class and additional pertinent parameters. Our analysis revealed that the default option yielded the most optimal performance by providing the most accurate representation of the current distribution without causing the model to overfit (see Merow et al. [20] for details). We restricted the background point number to 10,000 during sampling. However, a further increase in background point number (e.g., 100,000) made no difference to the model. The maximal iteration number was set to 1,000, which provided sufficient time for model convergence, and a convergence threshold was selected at 1 × 10^− 6^ [8, 45]. We employed the standard ‘autofeatures’ setting, encompassing all possible features such as linear, quadratic, product, threshold, and hinge characteristics (following Deb et al. [43])

Response curves were used to interpret Maxent output patterns. A Jackknife test was performed to analyze the relative importance of the environmental variables. The robustness of the Maxent model was calibrated using tests of threshold-independent receiver operating characteristics (ROCs). The area under the ROC curve (AUC) and True Skill Statistics (TSS) were used to evaluate the model performance. TSS is a threshold-dependent evaluation metric used to assess the performance of species distribution models. It ranges from − 1 to + 1, where + 1 indicates perfect agreement between observed and predicted presences and absences, 0 indicates a performance no better than random, and − 1 indicates total disagreement. TSS is particularly useful in cases where presence-only data is available, as it does not require information about true absences. It considers both sensitivity (true positive rate) and specificity (true negative rate) and is less affected by prevalence than other metrics like accuracy. Four types of potential habitats were developed based on the final potential distribution map, with values ranging from 0 to 1. The habitat was classified as highly suitable (> 0.75), moderately suitable (0.50–0.75), poorly suitable (0.25–0.50), or as providing no potential habitat (< 0.25), following the literature by Coban et al. [46] and Adhikari et al. [47]. Moreover, by comparing this data with the currently known suitable habitat, future potential distribution maps were re-categorized as, (i) becoming suitable, (ii) becoming unsuitable, and (iii) remaining suitable; the spatial extents of areas in each category were calculated and are presented.

## Data Availability

All relevant data can be found within the manuscript.
